# Nitrogen nutrition is a key modulator of the sugar and organic acid content in citrus fruit

**DOI:** 10.1371/journal.pone.0223356

**Published:** 2019-10-10

**Authors:** Ling Liao, Tiantian Dong, Xia Qiu, Yi Rong, Zhihui Wang, Jin Zhu

**Affiliations:** 1 College of Horticulture, Sichuan Agricultural University, Chengdu, China; 2 Institute of Pomology and Olericulture, Sichuan Agricultural University, Chengdu, China; 3 Sichuan Provincial Agricultural Department, Chengdu, China; Universidade Federal de Vicosa, BRAZIL

## Abstract

‘Huangguogan’ (*Citrus reticulata* × *C*. *sinensis*) is a new cultivar of mandarin citrus in China, and the research on fertilization of ‘Huangguogan’ is very limited. In this study, the effect of N fertilization on ‘Huangguogan’ fruit quality was determined at ripening. Sugars (sucrose, fructose, and glucose), organic acids (pyruvic, oxalic, citric acid, etc.), and vitamin components were measured at six stages of fruit development, and eight enzymes related to the glycolytic and Krebs cycle were assessed. The 1.81 kg N y^-1^ treatment group showed the highest total soluble solids concentration and total soluble solids/titratable acidity ratio but the lowest titratable acidity (acid content) at ripening, while the N_1_ treatment (0 kg N y^-1^) showed the opposite trend. Sucrose and citric acid accumulated to the largest extent during fruit development. Sucrose and ascorbic acid content increased (8.46 to 50.97 mg g^-1^ and 8.16 to 27.39 mg g^-1^, respectively), while citric acid content decreased (90.81 to 0.02 mg g^-1^). Aconitase was the key enzyme responsible for the observed changes in citric acid. The N concentrations in ripening fruit ranged from 2.25% to 4.15%. Curve estimation and principal component analysis revealed that fruit N was positively correlated with the sugars and vitamin components and negatively correlated with the organic acids. The accumulation of these metabolites seemed closely related to the dynamic changes in fruit N concentration at the five N levels tested. In conclusion, we suggest that the 1.81 kg N y^-1^ treatment represents the most suitable N fertilizer treatment for ‘Huangguogan’ citrus fruit.

## Introduction

Citrus fruit is a great source of naturally occurring nutrients, such as sugars, organic acids, and vitamin C, which are beneficial and important components of a healthy diet [1, 2]. Citrus fruit development can be divided into different stages: cell division, expansion phase, and ripening phase [3, 4]. Development of fruits is usually accompanied by sugar accumulation and organic acid degradation [2, 5]; e.g., sucrose levels are higher in orange juice when compared with the whole fruit [6]. Three important carbohydrates, namely, fructose, glucose, and sucrose, are usually found in a 1:1:2 ratio in whole fruit [7]. In contrast to the changes in sugar metabolism, organic acids usually accumulate during the early stages of fruit development and decrease during fruit ripening and storage [8, 9]. Citric and malic acids are the most abundant organic acids found in both climacteric and non-climacteric ripe fruits [2, 10]. Sugar accumulation during fruit maturation depends on sucrose accumulation [11], which in turn depends on the enzymes involved in sucrose metabolism, namely sucrose phosphate synthase (SPS) [12], sucrose synthase (SS) [13], acid invertase (AI), and neutral invertase (NI) (S1 Fig). High acid content often reduces fruit quality, but a moderate concentration of acid can improve the palatability of fruits. Organic acids also play a crucial role in food nutrition and processing [14, 15]. The acid content of fruit is determined by the balance of acid synthesis and degradation; e.g., the degradation of citric acid is catalyzed in the cytosol by enzymes such as phosphoenolpyruvate carboxylase (PEPC), citrate synthase (CS), and aconitase (ACO) [8, 16–18] (S2 Fig). Vitamin C (ascorbic acid) is one of the most vital water-soluble vitamins that naturally occurs in various fruits and vegetables and which cannot be synthesized by human bodies [19, 20]. Ascorbic acid (AsA) is a key co-factor for various enzymes, and participates in the regulation of photosynthesis, hormone biosynthesis, and plant senescence [21]. The total ascorbic acid content (T-AsA) of citrus fruit is the sum of AsA and dehydroascorbic acid (DHA) [22]. Several efforts have been made to enhance AsA levels in various fruits [23–25].

Nitrogen (N) is a key element that plays various roles in the life cycle of plants, i.e., it is an important constituent of chlorophyll in plants and it is the main nutrient involved in the synthesis of amino acids [26, 27]. N supply affects the content of some sugars, amino acids, and AsA [28, 29], which can influence the taste and flavor of fruits. ‘Huangguogan’ (*Citrus reticulata* × *C*. *sinensis*) is a new cultivar of mandarin citrus in China. The phenological period of Huangguogan involves early florescence in early March, the blossom period in mid-late March, the first physiological fruit drop period in mid-April, the second physiological fruit drop period in early May, the fruit expansion period from late June to early July, the fruit color change period in November, and the fruit ripening period from March to May the following year. The fruit is characteristically late maturing, high-yielding, seedless, and strong flavored; all of which favor the marketing potential of this cultivar [30]. However, its high fruit acidity at harvest make it less acceptable. Many studies [31, 32] have reported the effect of N fertilizer on citrus quality, however, few studies have explored the association between fruit quality and fruit N concentration at ripening in response to N fertilization [33]. Our previous studies showed that the rational application of phosphorus and potassium fertilizer can increase the sugar content and reduce acid content of Huangguogan [34] The use of the trace of ^15^N element when applying N fertilizer in the key phenological stage can improve the N absorption of trees [35]. We speculated that the rational application of N fertilizer during the key phenological period could increase the sugar content (quality) and decrease the acidity of Huangguogan. The aim of the present study was to investigate the effect and underlying mechanisms of N fertilization on Huangguogan fruit flavor, by assessing the soluble sugar, organic acid, and ascorbic acid content. Previous studies [36, 37] assessed the influence of N on the activities of key enzymes that are directly related to sucrose and citric acid metabolism in citrus fruit juice during fruit development and ripening. These enzymes were determined to be SPS, SS, AI, NI, PEPC, CS, and ACO, and thus we investigated these specific enzymes in the present study. In addition, we measured the effects of different nitrogen rates on yield and quality of ‘Huangguogan’ fruits. The changes rules of sugars, organic acids, and vitamin components should provide a useful us to understand the metabolic pathways underlying developmental in plants grown under different conditions of nitrogen supply. On the basis of the results obtained, we propose an optimal nitrogen application rate for best seedling growth of the citrus cultivar ‘Huangguogan’.

## Materials and methods

### Plant material and growth conditions

Field studies were conducted from March 31, 2017 to March 31, 2018 in Shimian County, Sichuan Province, China (29.23°N, 102.36°E; 780 m above sea level). Twenty-five 10-year-old, healthy ‘Huangguogan’ trees with trifoliate orange (Poncirus trifoliata) for rootstock were selected and divided into five N-treatment groups that each comprised five replicates. Individuals from each treatment group were planted in 5 m rows, spaced at 6 m from each other, in a sandy loam soil (Table 1). All plants were fertilized using 1.45 kg phosphorus (CaP_2_H_4_O_8_, P_2_O_5_ ≥ 12%) and 2.12 kg potassium fertilizer (K_2_SO_4_, K_2_O ≥ 50.0%) per year. N fertilizer [CO(NH_2_)_2_, N ≥ 46.67%] was applied as follows: 0 (N_1_), 1.36 (N_2_), 1.81 (N_3_), 2.26 (N_4_), or 2.72 (N_5_) kg y^-1^ at the germination stage of spring shoot (G), physiological fruit dropping (P), young fruit expansion (Y), and color-change stage (C), respectively, and in the ratios of G:P:Y:C = 40:10:40:10% for CO(NH_2_)_2_ application, 30:10:40:20% for CaP2H4O8 application, and 20:30:40:10% for K_2_SO_4_ application. Fertilizer was applied to each tree tray, i.e., at least 5 m apart in any given direction, which was a sufficient amount of spacing to ensure that the nitrogen supply did not diffuse to the adjacent trees. All trees received normal horticultural care for pest and disease control throughout the experiment.

**Table 1:** The main chemical and physical proprieties of the soil in the study area.

Index	Organic Matter (g kg^-1^)	Total N (mg kg^-1^)	Hydrolytic N (mg kg^-1^)	Available P (mg kg^-1^)	Available K (mg kg^-1^)
Content	26.63	0.96	90.17	46.73	66.94

### Fruit sampling

Fruit samples were drawn from each tree subjected to the various N treatments at six key fruit developmental stages, i.e., 60, 120, 180, 240, 300, and 360 days after the first blossom (DAFB). Overall, 40 representative fruits were collected from the north, east, south, and west sides of each replicate. Fruit samples were transported to the laboratory, and the pulp was quickly separated and evenly divided into three parts: one part was frozen in liquid N and stored at -80°C for sugar, organic acid, and key enzymes determination; the second part was weighed and then squeezed for fruit juice, which was filtered and then used to determine the fruit quality, including total soluble solid (TSS), titratable acidity (TA), and the TSS/TA ratio (RTT); the third portion of each fruit sampled was used to determine the N content using a Kjeldahl Auto Analyzer (K9840, HANON, China).

### TSS, TA, and yield measurement

The TSS was measured using a hand-held refractometer (Atago Co. Ltd., Japan), and TA (mEq NaOH 100 g/fresh fruit) was measured using 10 mL fruit juice diluted with distilled water (1:2) and titrated to pH 8.2 using 0.1 N NaOH. RTT was calculated as the ratio of total soluble solids and titratable acidity [38]. We weighed the fruit (g) using an AL204 precision electronic balance (Sartorius AG, Germany) and recorded the biomass yield per plant.

### Sugar and organic acid extraction and high-performance liquid chromatography (HPLC) analysis

Modified from Chen [5], 2 g samples of frozen pulp were ground to a powder in liquid N, homogenized in 5.0 mL of ethanol (80%) solution, and placed in a water bath at 80°C for 15 min. The homogenates were centrifuged at 9,000 × g for 10 min, at 4°C. The residues from each treatment were extracted in triplicate and the supernatant of each extracted residue was collected in a 10 mL volumetric flask and made up to the final volume (10 mL) with 80% ethanol. Samples were filtered through a 0.45 μm pore size membrane filter before injection into the HPLC system. Each sample was injected three times. The filtered solution was used for sugar and organic acid analysis.

#### HPLC analysis of sugars

Acetonitrile: water (80:20) was used as the mobile phase with a flow rate of 1.0 mL min^-1^. The column was Innoval NH_2_ (4.6 × 250.0 mm, 5.0 μm) (Bonna-Agela, Inc., China); eluted peaks were detected using a refractive index detector RI-1530 (Jasco Corp., Japan). Quantification of individual sugars was made by comparison with peak areas of standard sugars.

#### HPLC of organic acids

The column was WatersC_18_ (4.6 × 250.0 mm, 5.0 μm) (Waters technology (Shanghai) co., LTD, China). The flow rate was 0.8 mL min^-1^ using 3% methanol solution as the solvent. The methanol was adjusted to pH 2.6 using H_3_PO_4_. Organic acids were detected at a wavelength of 210 nm. The eluted peaks were detected with a Waters2996 diode array detector (Waters technology (Shanghai) Co., LTD, China), and quantification of individual organic acids was made using peak areas of standard acids.

### Total ascorbic acid extraction and quantification

The T-AsA was extracted and quantified following Enriqueta *et al*. [39], with some modifications. Briefly, 0.5 g of tissue was homogenized for 1 min using a Polytron homogenizer with 0.2% metaphosphoric acid (6 mL). The homogenate was centrifuged for 15 min at 12,000 × g at 4°C. The extract was subsequently filtered through a 0.22 μm filter membrane. Filtrate (1 mL) was injected in the HPLC system for AsA determination. Filtrate (3 mL) was added to 10 mL 0.2 mol L^-1^ dithiothreitol and, after dark treatment for 4 h, was used to determine the T-AsA, and DHA = T-AsA–AsA.

#### HPLC of AsA

The supernatant was filtered through a WatersC18 (4.6 × 250.0 mm, 5.0 μm) column (Waters technology (Shanghai) Co., LTD, China). Methanol: 2% metaphosphoric acid (15:85) was used as the mobile phase with a flow rate of 1.0 mL min^-1^.

### Enzymes related to sugar and organic acid metabolism measurement

Eight enzymes were assessed in this study: SPS, SS (both cleavage and synthesis direction), AI, NI, PEPC, CS, and ACO. Frozen samples were ground in liquid N and weighed to 0.05–0.10 g. All enzymes were extracted in an ice-bath and determined using ELISA kits (Suzhou Comin Biotechnology, Co., Ltd, China), following the instructions of the manufacturer.

### Statistical analysis

The results were expressed as means ± standard errors (SEs). A one-way analysis of variance (ANOVA) was conducted on data and the differences between means were determined by Duncan test at p < 0.05 using the IBM SPSS Statistics 20.0 analytical software. Figures were prepared using the Origin 8.0 (Origin Lab) software.

## Results

### Nitrogen is a determinant element that drives citric fruit quality

**Fig 1:**
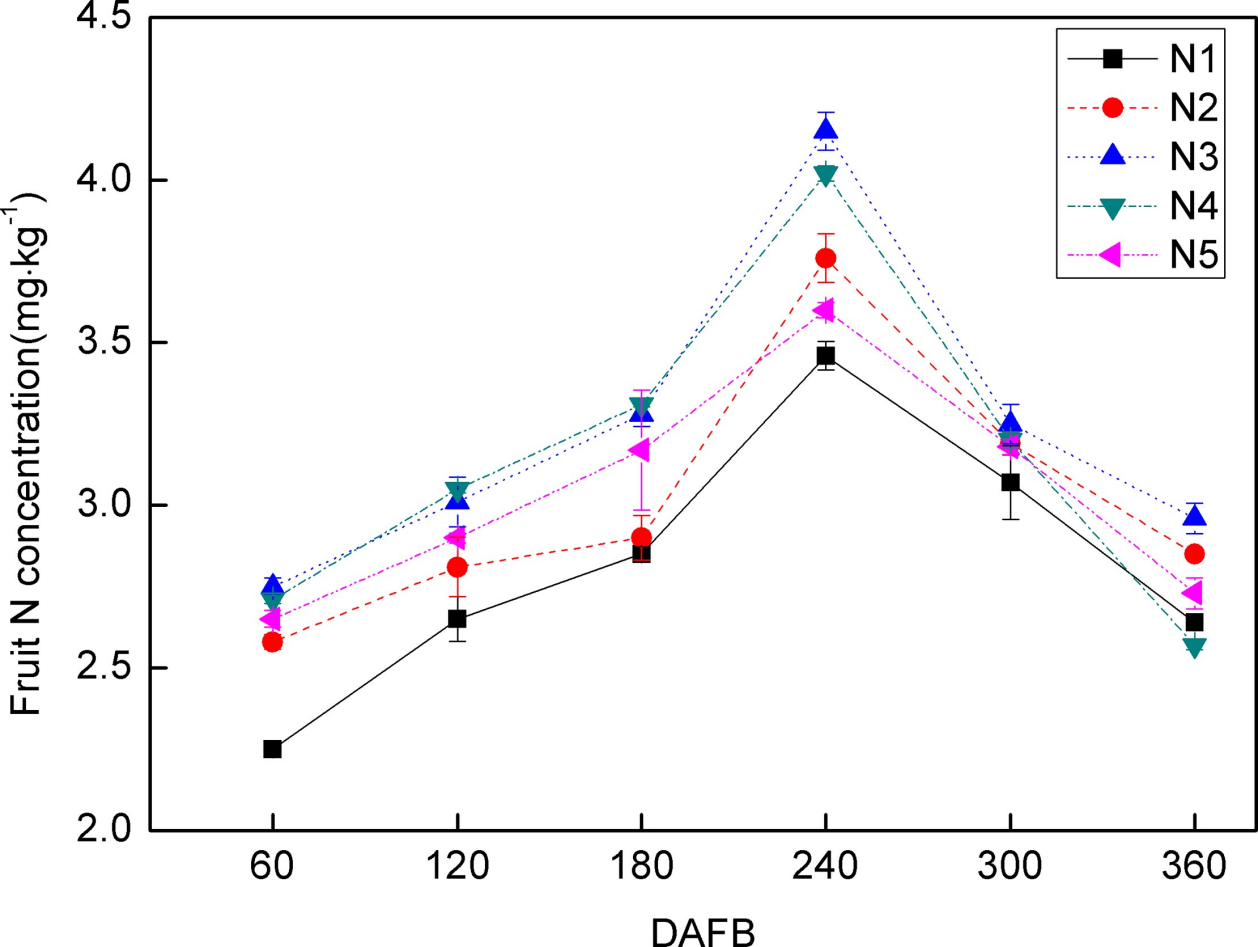
Dynamic changes in N concentration in ‘Huangguogan’ fruit during fruit development in response to five N fertilizer levels: 0 (N_1_), 1.36 (N_2_), 1.81 (N_3_), 2.26 (N_4_), or 2.72 (N_5_) kg y^-1^. Error bars indicate the standard deviation of six replicates.

In the present study, N was observed to accumulate in the fruit pulp from 60 DAFB, peak at 240 DAFB, and then decrease until 360 DAFB. The ranges in fruit N concentration in response to the various N fertilization treatments were: N_1_ (2.25–3.46 mg kg^-1^), N_2_ (2.58–3.76 mg kg^-1^), N_4_ (2.71–4.02 mg kg^-1^), and N_5_ (2.65–3.60 mg kg^-1^) from 60 to 180 DAFB, which were not as great as N_3_ (2.75–4.15 mg kg^-1^) (Fig 1).

The TSS, TA, RTT, yield, and single-fruit weight were measured at the ripening stage (Table 2). Our findings indicated that the flavors differed among the five N treatment groups of Huangguogan fruit. Significant differences in fruit quality were observed among the pulp juices of the five N treatment groups of Huangguogan fruit at 360 DAFB. Treatment N_3_ showed a much higher TSS concentration (13.07°Bx) and TSS/TA ratio (18.66) than the other treatments, and a lower TA concentration (0.69%) than the other treatments. In contrast, treatment N_1_ showed a remarkably higher TA concentration (0.91%), but lower TSS concentration (12.43°Bx) and TSS/TA ratio (13.67) than the other treatments. It is also noteworthy that the TSS and TA were decreased in the N4 and N5 treatment groups., although the decrease was not found to be significant compared to N_1_ (*P* > 0.05).

**Table 2:** ‘Huangguogan’ fruit quality at the ripening stage in response to five N levels: 0 (N_1_), 1.36 (N_2_), 1.81 (N_3_), 2.26 (N_4_), or 2.72 (N_5_) kg y^-1^ during growth.

Treatment	N_1_	N_2_	N_3_	N_4_	N_5_
TSS (%)	12.43 ± 0.38 ^a^	12.93 ± 1.18 a	13.07 ± 0.57 ^a^	12.27 ± 1.17 ^a^	12.03 ± 1.10 ^a^
TA (g 100 mL^-1^)	0.91 ± 0.04 ^a^	0.70 ± 0.03 ^b^	0.69 ± 0.01 ^b^	0.67 ± 0.04 ^b^	0.66 ± 0.03 ^b^
RTT	13.67 ± 0.99 ^b^	18.49 ± 1.56 ^a^	18.66 ± 1.40 ^a^	18.42 ± 1.39 ^a^	18.08 ± 2.38 ^a^
Yield (kg plant^-1^)	103.67 ± 2.22 ^d^	144.07 ± 4.71 ^b^	158.02 ± 10.95 ^a^	136.57 ± 4.02 ^b^	127.77 ± 4.17 ^bc^
Single-fruit weight (g)	151.83 ± 1.50 ^a^	130.24 ± 2.89 ^c^	153.20 ± 2.55 ^a^	130.78 ± 2.43 ^c^	144.22 ± 2.42 ^b^

TSS, total soluble solids; TA, titratable acidity; RTT,_the TSS/TA ratio. Data are means ± SD of five replications. Different lowercase letters indicate significant differences (P < 5%) among treatments.

All the assayed N treatments produced high Huangguogan yields and high single fruit weights compared to N_1_. The yield and single fruit weight increased with the increase in N application, however, beyond a certain level, the excess N did not further produce an increase in production, as expected. As a result, the N_3_ treatment yielded statistical more fruit than N_4_ and N_5_ (Table 2), and the highest increase in yield and single fruit weights.

### Changes in sugar accumulation and organic acid degradation during fruit development

**Fig 2:**
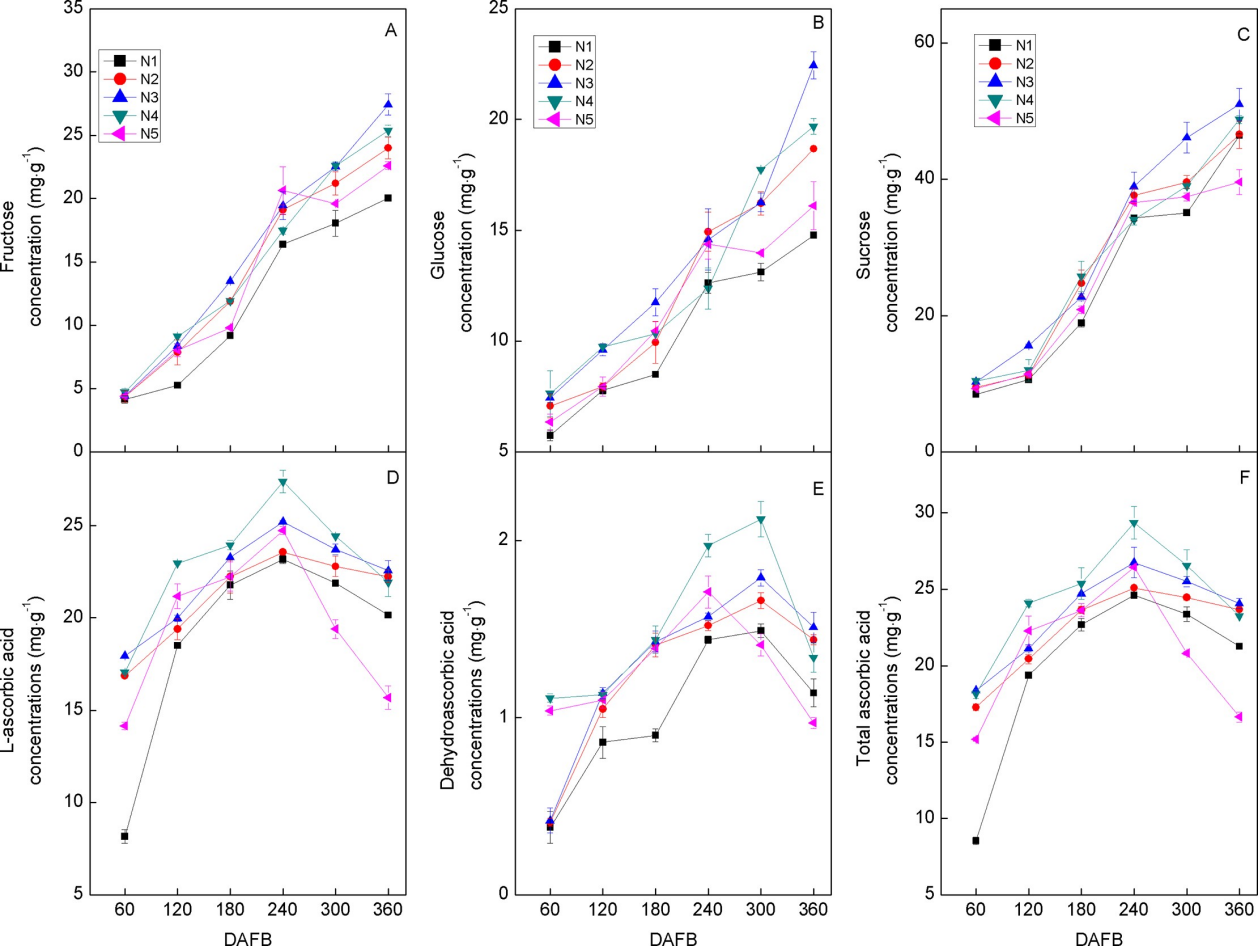
Dynamic changes in the concentration (mg g^-1^) of ‘Huangguogan’ fruit sugars (A–C) and VCs (D–F) during fruit development (days after the first blossom, DAFB) in response to five N fertilizer levels: 0 (N_1_), 1.36 (N_2_), 1.81 (N_3_), 2.26 (N_4_), or 2.72 (N_5_) kg y^-1^. Error bars indicate the standard deviations of six replicates.

Sucrose, glucose, and fructose were determined as the sugar components, and the variations in fruit sugar concentrations were the same in the five N treatment groups of Huangguogan, i.e., sugar components accumulated in the fruit pulp increased from 60 DAFB to 360 DAFB (Fig 2A–2C). The total or individual sugar concentrations were higher in the fruits in all N treatments and at all developmental stages than the N_1_ group. Sucrose was the major sugar component at every stage and ranged from 8.46 to 50.97 mg g^-1^. Fructose and glucose ranged from 4.13 to 27.43 mg g^-1^ and 5.75 to 22.44 mg g^-1^, respectively. We found that the concentrations of all sugar components increased with increasing N supply, and that the highest and lowest sugar components concentrations were observed in N_3_ and N_1_, respectively.

In the present study, accumulated ASA, DHA and T-ASA in the fruit pulp increased from 60 DAFB to 300 DAFB, followed by a slight decrease (Fig 2D–2F). The change in ASA, DHA, and T-ASA with the increase in N fertilizer was similar to that observed in the sugar content, whereby the highest and lowest sugar component concentrations were observed in N_4_ and N_1_, indicating that reduced vitamin components (VC) resulted from excess N supply.

**Fig 3:**
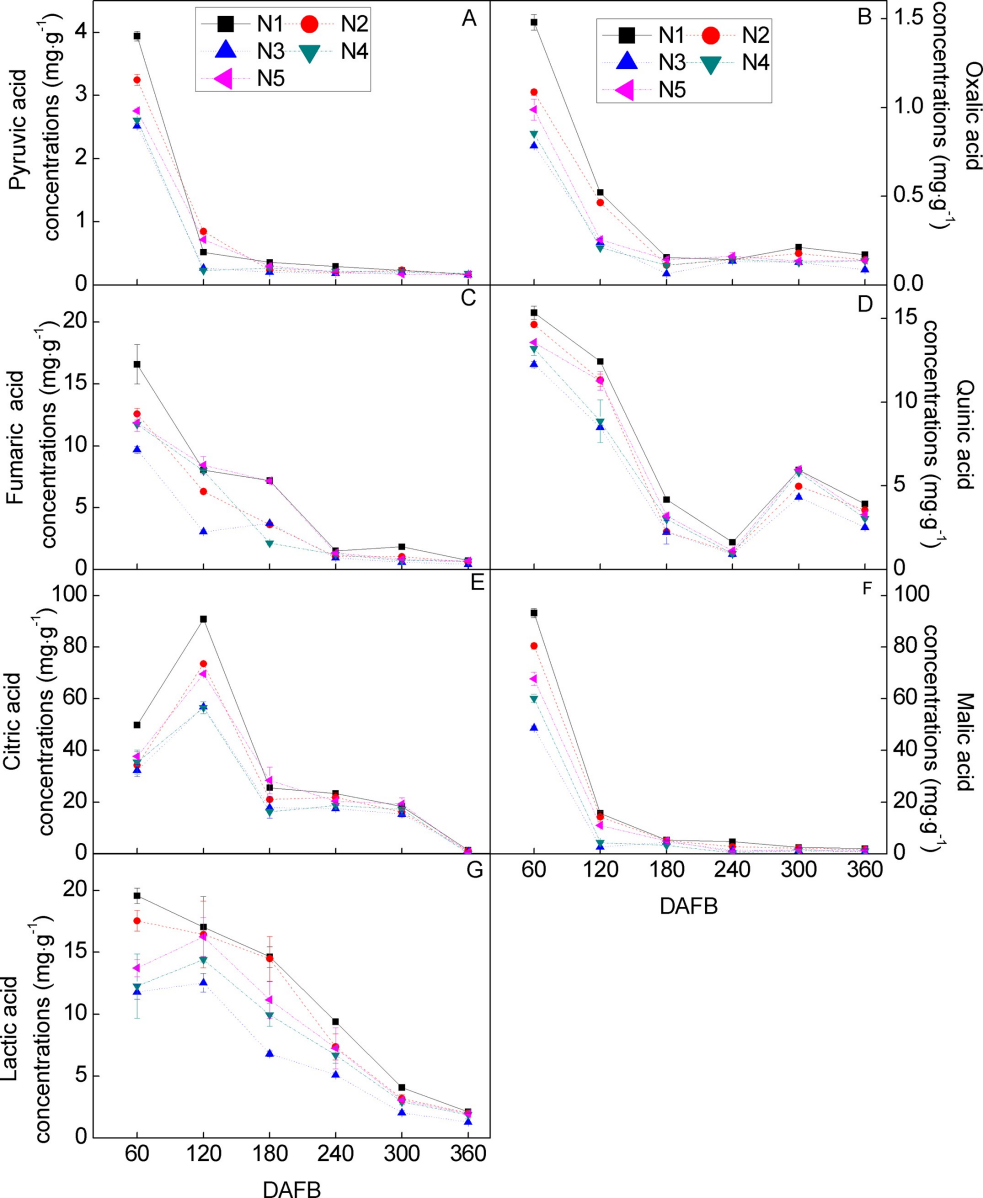
Dynamic changes in the concentration (mg g^-1^) of ‘Huangguogan’ organic acids during fruit development (days after the first blossom, DAFB) in response to five N fertilizer levels: 0 (N_1_), 1.36 (N_2_), 1.81 (N_3_), 2.26 (N_4_), or 2.72 (N_5_) kg y^-1^. Error bars indicate the standard deviations of six replicates.

Organic acids, including pyruvic, oxalic, fumaric, quinic, lactic, malic, and citric acid, were measured during Huangguogan fruit development and in response to N fertilization (Fig 3A–3G). The observed trends regarding fruit organic acids differed greatly from those of the sugar content, whereby the citric acid content, which was found in higher concentrations than the other organic acids, decreased during fruit growth and ripening. Compared to the citric acid level, the malic acid content was slightly lower, while that of pyruvic acid and oxalic acid were much lower. The concentration of organic acids was significantly higher in the samples from the N_1_ group than in the N treatment groups, and they were lowest in the N_3_ treatment.

### Citric acid abundance is determined by aconitase under good N availability

**Fig 4:**
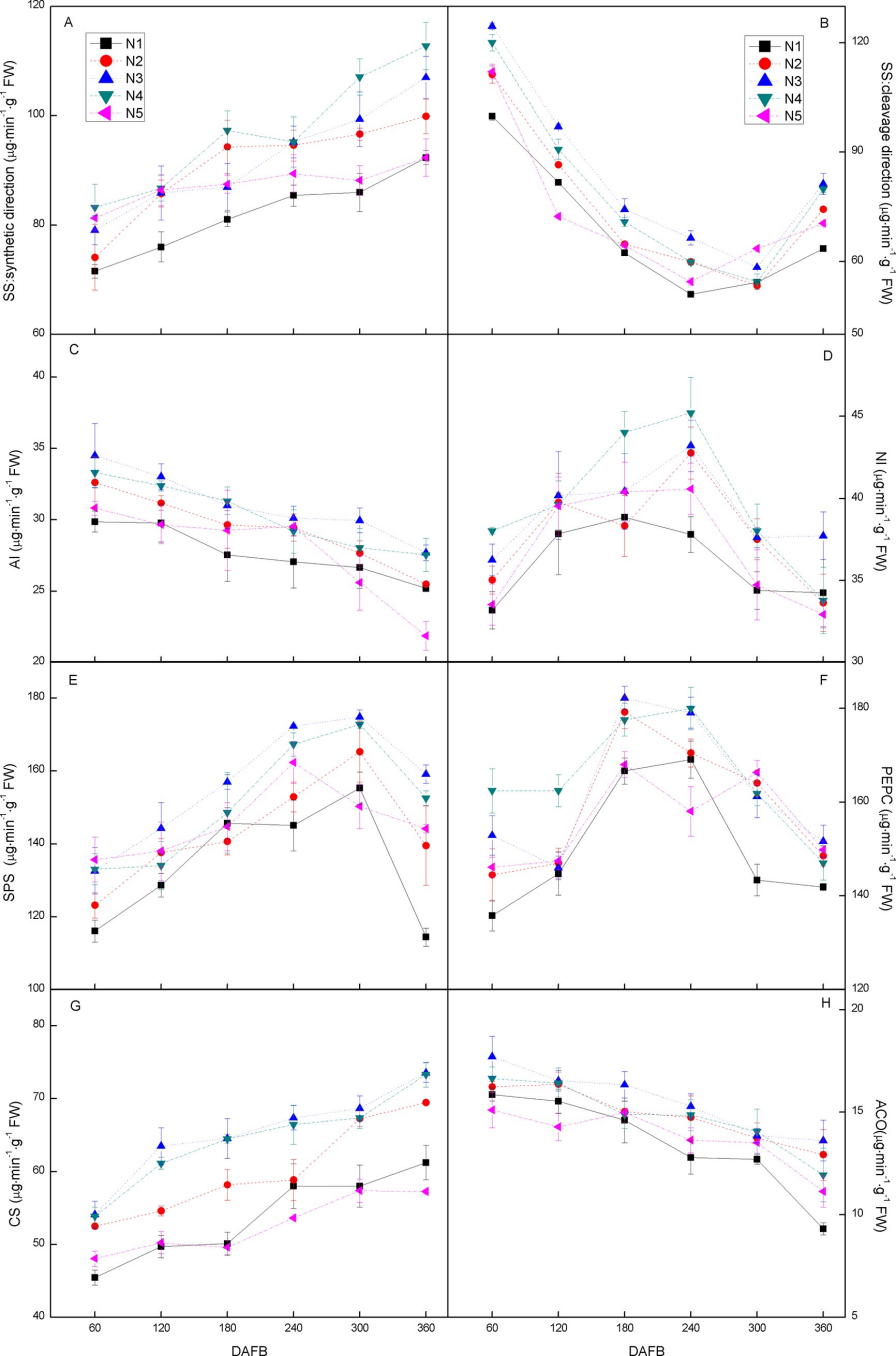
Dynamic changes in enzymes [sucrose synthase, SS (A, B); acid invertase, AI (C); neutral invertase, NI (D); sucrose phosphate synthase, SPS (E); phosphoenolpyruvate carboxylase, PEPC (F); citrate synthase, CS (G); and aconitase, ACO (H)] involved in glycolysis and in the Krebs cycles during fruit development (days after the first blossom, DAFB) in response to five N fertilizer levels: 0 (N_1_), 1.36 (N_2_), 1.81 (N_3_), 2.26 (N_4_), or 2.72 (N_5_) kg y^-1^. Error bars indicate the standard deviations of three replicates. Fw, fresh weight.

Among the enzymes related to sugar metabolism, we found higher activities of SPS and SS in both two directions than of AI and NI. In addition, SPS and SS in the synthetic direction exhibited opposite dynamic changes to the activities of AI and NI. SS in the cleavage direction decreased from 60 to 180 DAFB, then increased until 360 DAFB. The activities of SPS, SS (both cleavage and synthetic direction), AI, and NI increased significantly in response to the N treatments compared to the N_1_ (Fig 4A–4E). Our results showed that PEPC activity increased during the initial fruit development stages and then decreased gradually (Fig 4F), while CS increased continuously as the fruit developed and matured (Fig 4C). The activities of ACO decreased continuously as the fruit developed and matured (Fig 4G). Meanwhile, the average level of CS activity was much higher than that of ACO. Moreover, the activities of the three enzymes (Fig 4F–4H) decreased significantly in response to the N treatments compared to N_1_.

### Relationships between fruit sugars, organic acid components, and N concentration during fruit development

**Fig 5:**
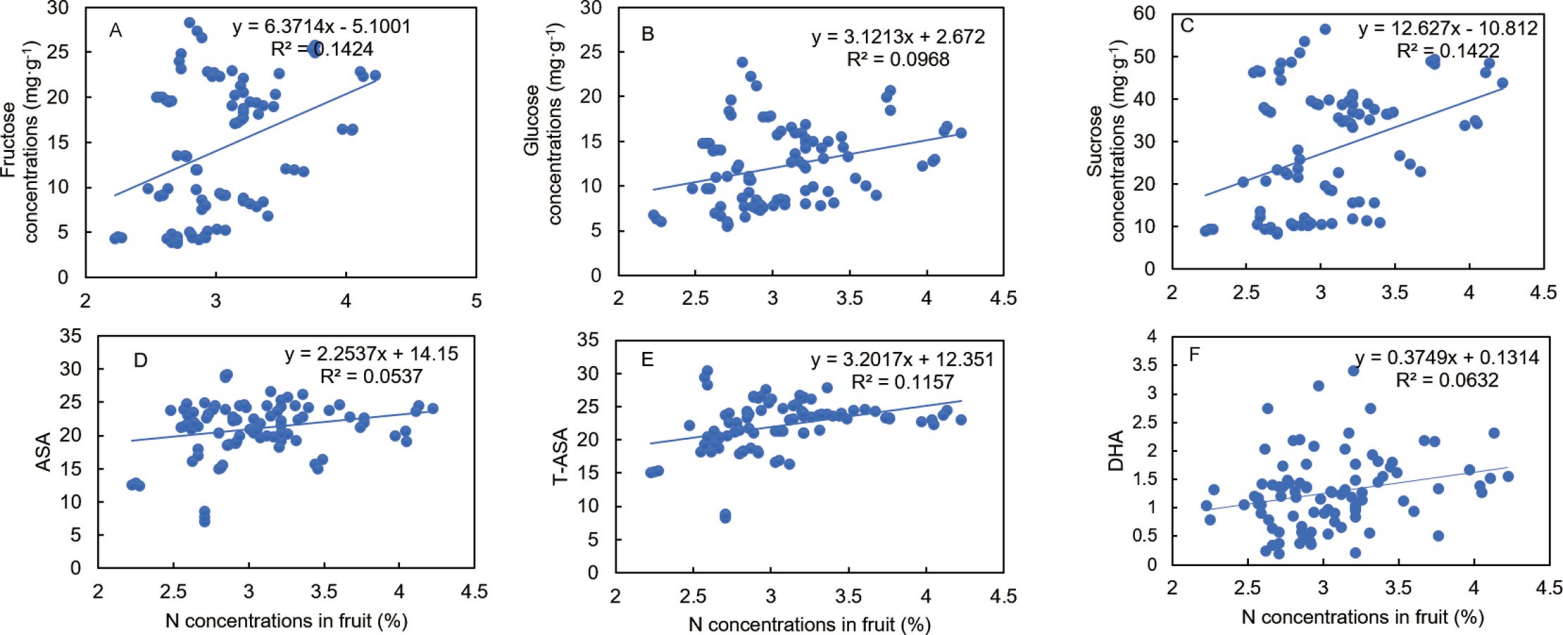
Response curves of ‘Huangguogan’ fruit N concentrations versus different sugars [fructose (A), glucose (B), sucrose (C)] and vitamin components [ascorbic acid, AsA (D); total AsA, T-AsA (E); dehydroascorbic acid, DHA (F)].

**Fig 6:**
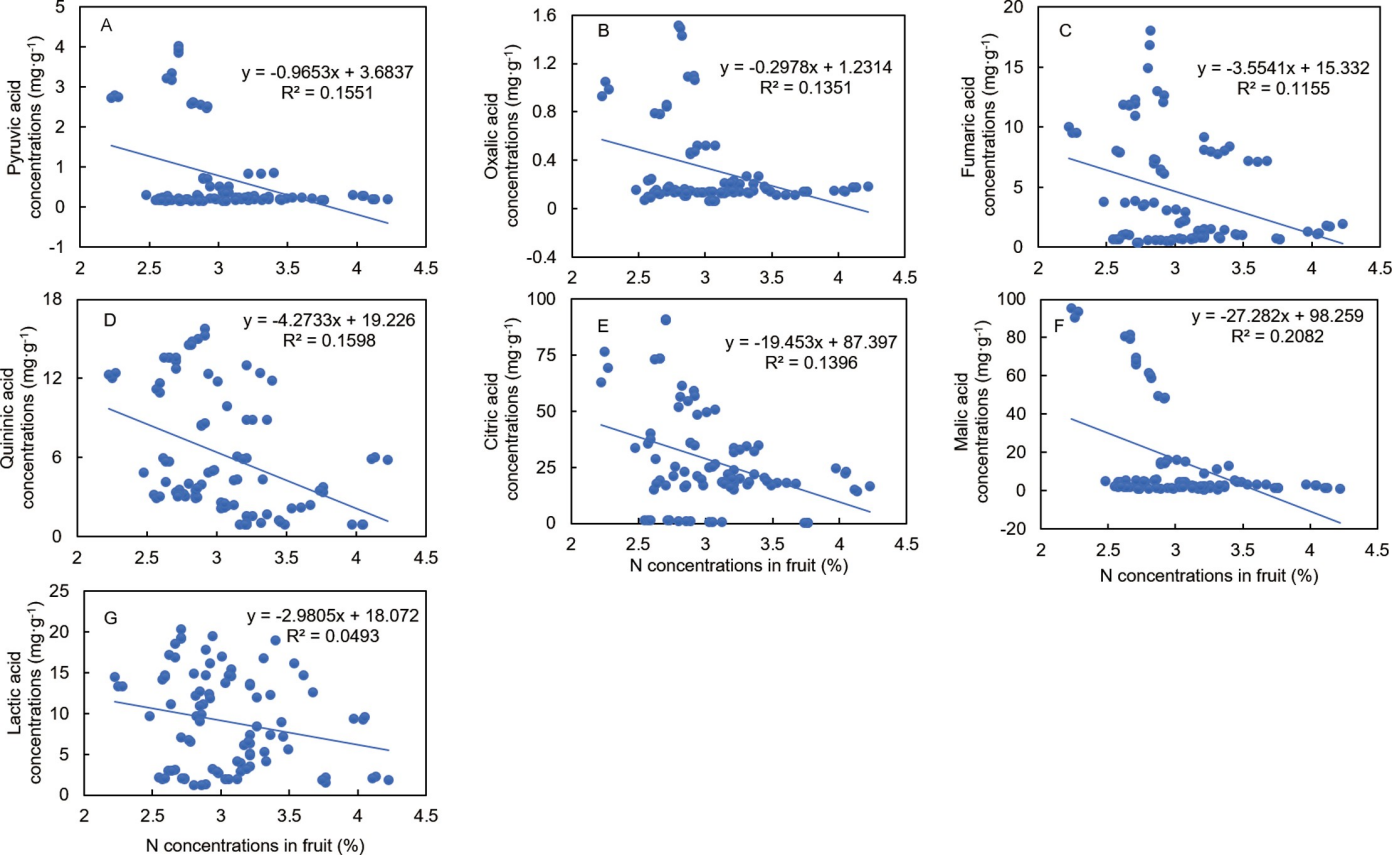
Response curves of ‘Huangguogan’ fruit N concentrations versus different organic acid components: (A) pyruvic acid, (B) oxalic acid, (C) fumaric acid, (D) quinic acid, (E) citric acid, (F) malic acid, and (G) lactic acid.

Curve estimation and PCA analysis revealed that fruit N concentration was correlated with fruit sugars, organic acids, and VC concentrations. The curves indicated that the higher the N concentration, the higher the sucrose, fructose, and glucose concentrations (Fig 5A–5C), i.e., there was a positive correlation between N concentration and sugar components. These results implied that N might favor sugar accumulation in fruit. Similarly, the curves indicated that the higher the N concentration, the higher the AsA, DHA, and T-AsA concentrations (Fig 5D–5F), i.e., N concentration is positively correlated with the VCs. These results implied that N might favor VC accumulation in fruit. On the other hand, there was a negative correlation between the organic acid components and N concentrations, whereby the higher the N concentration, the lower the organic acid concentration. These results indicated that N in fruit might favor organic acid degradation (Fig 6A–6G).

**Fig 7:**
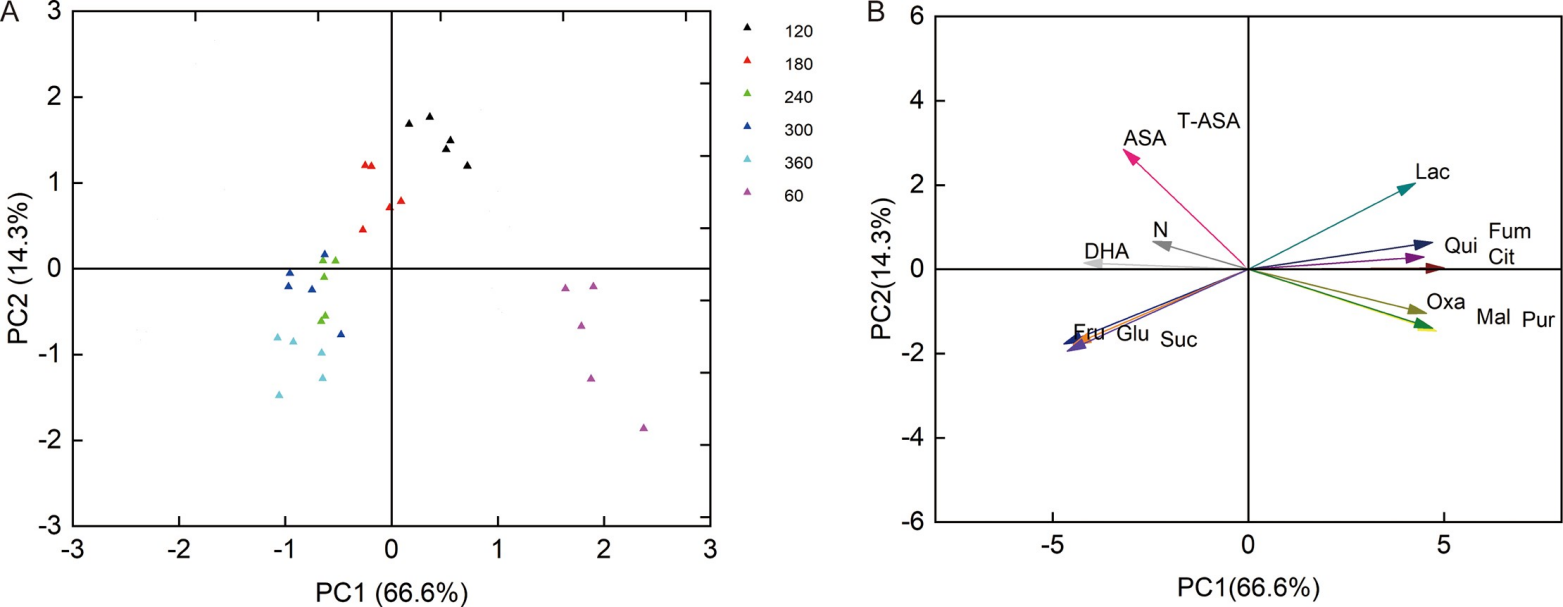
PCA analysis of sugars (fructose, Fru; glucose, Glu; sucrose, Suc), vitamin components (ascorbic acid, AsA; total AsA, T-AsA; dehydroascorbic acid, DHA), organic acids (pyruvic acid, Pur; oxalic acid, Oxa; fumaric acid, Fum; quinic acid, Qui; citric acid, Cit; malic acid, Mal; and lactic acid, Lac), and N in ‘Huangguogan’ fruits. (A) Score plot of PC2 against PC1 (n = 30); days after the first blossom, DAFB. (B) Load plot of PC2 against PC1 (n = 30).

As shown by the PCA analysis (Fig 7A and 7B), the five N fertilization treatments tested and the different fruit developmental stages were almost completely separated from each other (Fig 7A). Furthermore, there was an obvious relationship between the sugars, organic acids, VCs, and N (Fig 7B). Acute angles were found between the vector arrows of sugar components and N, and between the vector arrows of the VCs and N. However, blunt angles were found between the vector arrows of the organic acid components and N. These findings indicated that N was positively correlated with the sugars and VCs but negatively correlated with the organic acids (Fig 7B). Thus, the results of the curve estimations and PCA analysis were consistent.

## Discussion

Soil N should be available during the crop growth cycle to ensure high yields and maximum income [40]. Studies have demonstrated that the application of N fertilizer increases yield and enhances the quality of citrus [41]. This study showed that the total yield of Huangguogan increased with the increase in N application until the N_3_ treatment and then slightly decreased, these results agree with previous experiments performed on tomatoes [42], melon [43], and date palm [44] yield and N fertilization. In fruit trees, N is a major limiting factor on nutrition and reproductive growth, physiological and biochemical processes, as well as fruit yield and quality [45]. Plant growth has been shown to require the largest quantities of N [46]. N application can increase the amount of N in fruits [47]. Our results showed that appropriate application of N could increase the fruit N, but excessive application of N reduced the fruit N; thus, these results suggest that a high N fertilization level serves as a growth limiting factor. Fruit flavor is one of the most important traits of fruit quality. The key factors that affect consumer acceptance are principally governed by the levels as well as the ratios of sugars and organic acids [48]. TSS is the main index of sugar content in fruit [49]; citrus ripening is characterized by sugar accumulation with acid decrease and the ratio of TSS/acidity is one of the determining criteria for citrus maturity [50]. Previous studies have shown that the TSS increases as the N rate increases [51, 52]. Our study showed that the TSS increased with the increase in N application until N_3_ and then slightly decreased, although this decrease was not significant compared with N_3_. The absence of significant differences might be due to the level of N not being highly excessive, and thus the TSS of citrus presumably does not increase indefinitely with the increase in N application. These findings indicated that the N fertilization treatments sufficiently nourished the plants and significantly increased production [53–55], however, beyond an optimal treatment level, excessive N fertilization had a negative impact [56].

Organic acids as well as soluble sugars contribute highly to the flavor and overall quality of citrus fruit [57] and N levels have been shown to greatly influence the sugar composition [58]. The present study shows that the higher the N fertilization, the higher the amounts of sucrose, glucose, and fructose. The acidity of citrus fruit is mainly due to the levels of citric and malate acid [59, 60], and the influence of N fertilization has the most obvious impact on citric acid levels [61]. Based on quantity, the most important organic acids in Huangguogan are citric acid followed by malic acid and lactic acid. In addition, smaller amounts of pyruvic, oxalic, fumaric, and quinic acid were present. The processes involved in these pathways and in the Krebs cycle are vital for the accumulation and degradation of fruit sugars and organic acids [1]. SS represented the direction of synthesis and SPS was the frequency detected to explain the sugar accumulation. SS, in the direction of cleavage, and two invertases (AI and NI) were determined to explain the degradation of sucrose [3]. Sucrose and citric acid contributed mainly to the TSS and TA concentration, respectively [62], we found that TSS was significantly enhanced by N treatment, Moreover, the SS activity of cleavage direction and AI increased under N treatment, Hence, we hypothesize that the enhancement of SS (cleavage direction) and AI activities is the main reason for the increase of TSS in the fruit segment since they increase the sink strength for sucrose partition into fruit [63]. On the other hand, fruit pulp acidity is directly related to citric acid content [64], which is determined by the balance of the activities of PEPC, CS, and ACO [65, 66]. Apart from sugar accumulation, citrus fruit acidity was also affected by N treatment, whereby fruit acidity was obviously decreased by N treatment. The activities of enzymes directly related to citric acid synthesis (PEPC and CS) and degradation (ACO) were also investigated [63]. Sadka [65] reported that the increased activity of CS and decreased activity of ACO contributed to the accumulation of citric acid. In addition to our findings on citric acid concentration (Fig 2E), the accumulation of citric acid was shown to be regulated by the ACO more than by CS [1, 67].

A positive correlation has been confirmed between AsA and sucrose and glucose [68], and our results supported this. AsA contributes to the antioxidant capacity of plant tissues [69], and in many crops, the enhancement of AsA concentration also extends the shelf-life ^[^^70^^]^. Previous studies [71, 72] reported an increase in AsA content in fruit that received higher N fertilizer treatments; conversely, Nagy [73] demonstrated that an increased N supply to citrus trees resulted in lower AsA content in the which indicates that the response of ASA to N fertilizer differs among different fruit trees. For Huangguogan, appropriate N application was shown to increase ASA content in the fruit. However, farmers usually apply excessive N fertilizer that is not proportional to crop yield [74, 75]. Takebe *et al*. [76] concluded that sugar content decreased slightly with excessive N fertilizer treatments. Under excessive N, the distribution of photosynthate to juice sacs decreased, and the activities of sucrose metabolic catabolism enzymes, including AI, NI, and SPS, increased [77], which promoted further metabolic consumption of sucrose and was not conducive to the accumulation of sucrose [78]. Similarly, Li *et al*. [79, 80] concluded that excessive N input cannot increase the yield, but will reduce the vitamin content. However, excessive N has been shown to increase the total fruit acidity [81] and decrease the ratio of sugar to acid [82], which supports the findings of our correlation analysis. However, the physiological and molecular mechanisms of N in the metabolic regulation of sugars, organic acids, and VCs are not well understood. Thus, the regulating mechanisms of N in citrus fruit sugar organic acid and VC metabolism require further investigation.

## Conclusions

The present study revealed significant differences in fruit quality in response to the five N treatments tested. Sucrose and citric acid were found to be the major sugar and organic acid components in ‘Huangguogan’ fruits, respectively. However, the accumulated levels of these components differed among the five N treatments. All N treatments led to high sucrose levels in young fruit until ripening; however, compared to the N_1_ treatment, each N treatment showed different rates of sucrose accumulation. The highest sugar accumulation rates were recorded for the N_3_ treatment. In contrast, all N treatments resulted in low organic acid content than that in the N_1_ treatment, with N_3_ resulting in the lowest value. These findings indicated that an increase in N fertilizer resulted in an increase in sugar and a decrease in acid content; however, it is also noteworthy that excess N treatment resulted in lower fruit quality. A difference in citric acid concentration was observed among the five N levels of the fertilizer treatments, which was mainly attributed to degradation enzymes and not to the enzymes responsible for citric acid synthesis. Different N treatments can affect the uptake of N nutrients by fruit to various extents. Our study showed that N fertilization benefits fruit N accumulation, that fruit N concentration positively affects fruit sugar and VC concentrations, but significantly negatively affects fruit organic acids. In conclusion, we suggest that the N_3_ treatment represents the most suitable N fertilizer treatment for ‘Huangguogan’ citrus fruit. However, the physiological and molecular mechanisms of N-regulated sugar and organic acid metabolism are not well understood. Thus, these mechanisms in citrus fruit sugar and organic acid metabolism require further clarification.

## Supporting information

S1 Fig Enzymes related to sucrose metabolism in citrus. (JPG)

S2 Fig Enzymes related to citrus acid metabolism. (JPG)
