# Supplementary figures and images for: Nitrogen nutrition is a key modulator of the sugar and organic acid content in citrus fruit
Source: PLoS One. 2019 Oct 10;14(10):e0223356. doi: 10.1371/journal.pone.0223356 (PMC6786551; doi:10.1371/journal.pone.0223356)

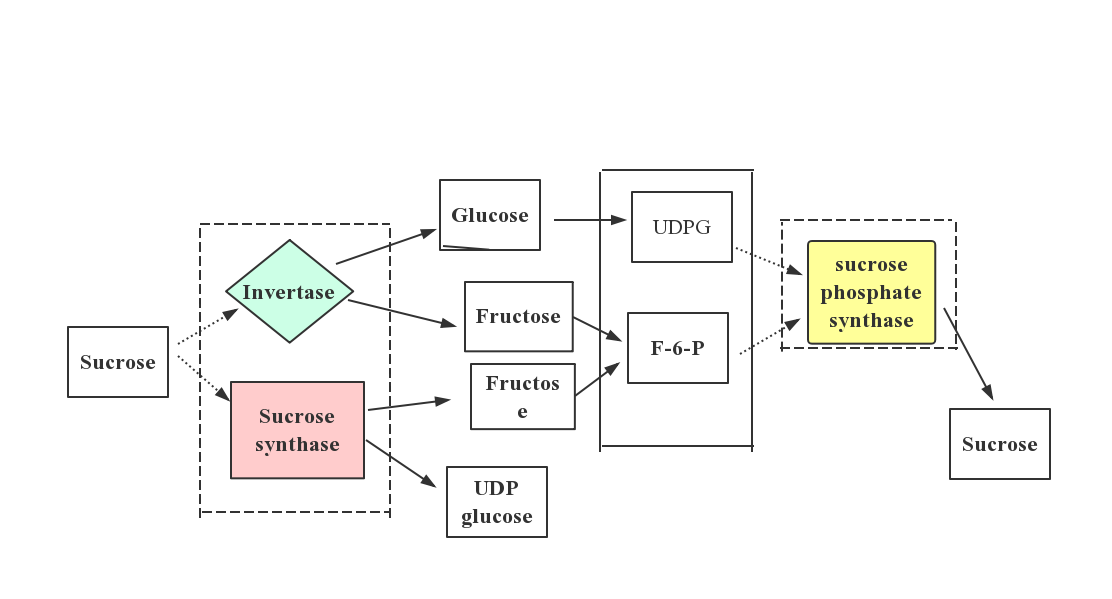

Supplement: S1 Fig — (JPG) [file pone.0223356.s001.JPG]

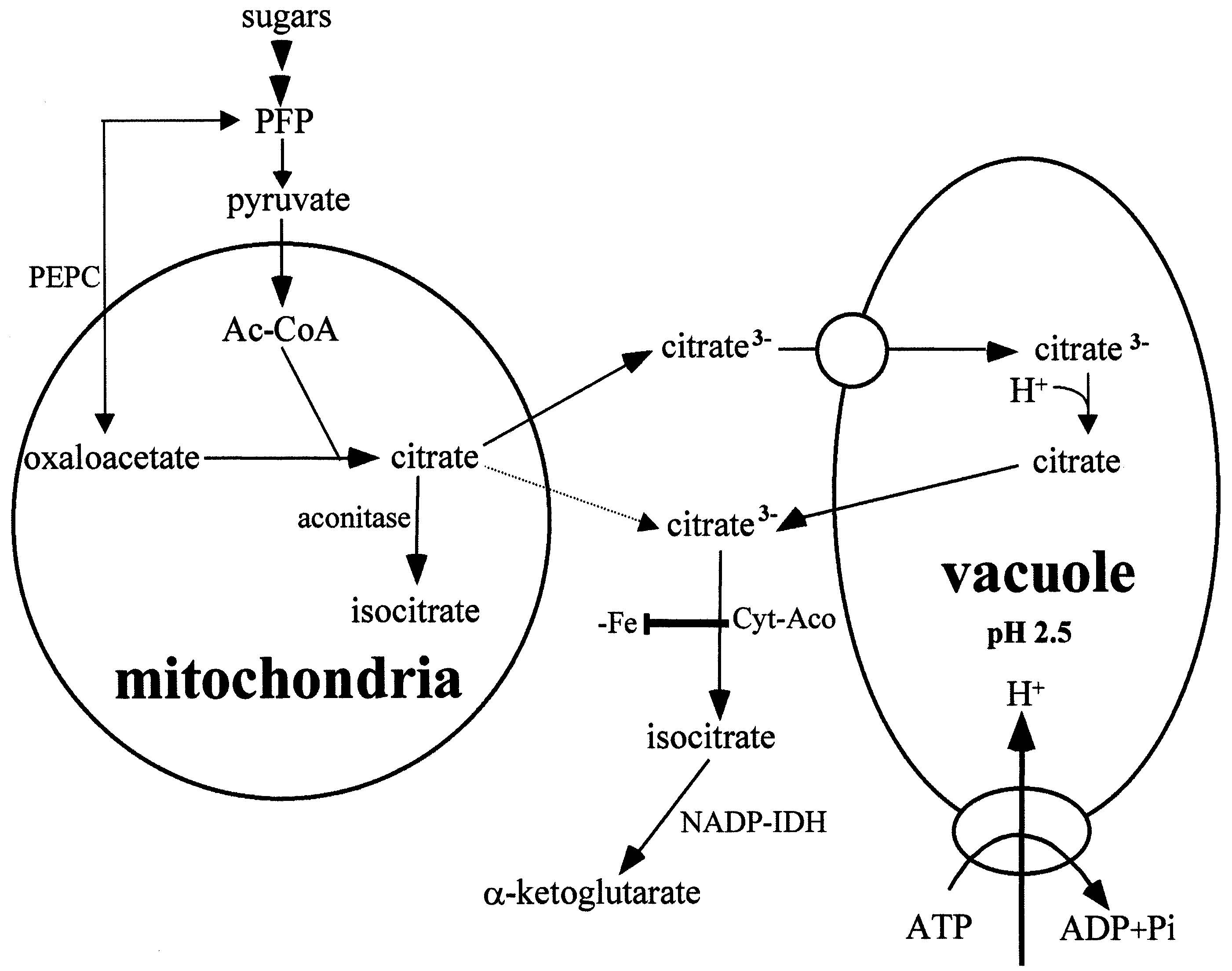

Supplement: S2 Fig — (JPG) [file pone.0223356.s002.jpg]
